# Lipid Management in Primary Care for Socioeconomically Disadvantaged Populations in Northern England: A Qualitative Study

**DOI:** 10.1177/21501319241272026

**Published:** 2024-10-19

**Authors:** Yu Fu, Sarah Sewdon, Julia L. Newton

**Affiliations:** 1 University of Liverpool, Liverpool, UK; 2 NIHR Applied Research Collaborative North East and North Cumbria, Cumbria, UK; 3 Northumberland and Tyne and Wear NHS Foundation Trust, Newcastle, UK; 4 Newcastle University, Newcastle upon Tyne, UK; 5 H﻿ealth Innovation, North East North Cumbria, Newcastle upon Tyne, UK

**Keywords:** primary care, lipid management, socioeconomically disadvantaged populations, qualitative study

## Abstract

**Introduction:**

People in low socioeconomic circumstances are more susceptible to dyslipidemia and cardiovascular disease than those living in more affluent populations. Limited healthcare access and low preventive care uptake widen health inequalities. Understanding how primary care can better serve socioeconomically disadvantaged communities is urgently needed.

**Aim:**

To explore lipid management delivery in socioeconomically disadvantaged areas and identify barriers and enablers for lipid optimization for socioeconomically disadvantaged populations.

**Method:**

Individual semi-structured remote interviews with clinicians, purposively recruited from primary care practices serving extremely socioeconomically disadvantaged communities in Northern England, UK, who were involved in the delivery and organization of lipid management. Interviews were recorded, transcribed, and analyzed thematically following framework analysis.

**Results:**

Fifteen interviews were undertaken. Five themes emerged: complex and multimorbid patients with competing priorities, limited access and follow-up to supporting services, being flexible and working beyond guidelines, high workload with inadequate staff support, and the need for care integrity and sustainable support.

**Conclusion:**

The findings of this study have been fed back to the delivery of the national program to improve cardiovascular health. Socioeconomically disadvantaged communities have complex health needs posing risks of multimorbidity but living with low health literacy, competing demands upon time, and financial constraints. Clinicians are willing to adapt services but a lack of guidance for care and funded services remains a significant barrier to targeted service delivery. Research is needed to inform the effectiveness and acceptability of interventions for lipid management tailored for those experiencing low socioeconomic disadvantage.

## Introduction

Unhealthy behaviors such as smoking, alcohol consumption, unhealthy diet, and physical inactivity are more prevalent in socioeconomically disadvantaged populations compared to more affluent populations.^1^ Individuals with lower socioeconomic status are more susceptible to dyslipidemia and have an increased risk of cardiovascular disease (CVD).^2,3^ Factors like income, education, employment, and environment contribute to socioeconomic deprivation, correlating with CVD incidence and outcomes in high-income countries.^4,5^ In the UK, CVD prevalence in the most deprived 10% of the population is twice as high as those in the least deprived,^6^ leading to a fourfold increased risk of premature death attributed to CVD in England’s most deprived areas between 2017 and 2019.^7^

The National Health Service (NHS) in England has committed to reducing heart disease disparities over the next decade.^8^ While interventions targeting behaviors, lifestyle, education, medication, and monitoring have been implemented to address health inequalities linked to low socioeconomic status, inconsistent outcomes persist in managing CVD risks.^9^ Risk assessment and lipid modification are pivotal in primary CVD prevention, yet an inverse care law exists in primary care,^10^ and limited access remains a barrier, particularly for socioeconomically disadvantaged populations.^4^ Notably, those living in socioeconomically disadvantaged are 20% more likely to develop CVD compared to affluent communities but are less likely to attend CVD screening from which they could greatly benefit.^11^ This indicates an urgent need to examine approaches and experiences of lipid management in the context of socioeconomic adversity to understand how primary care can better cater to the needs of individuals experiencing low socioeconomic disadvantage.

Over a third of the population in North East England live in the 20% most deprived areas, with a healthy life expectancy 4 years below the UK average.^12^ To address such inequalities, the Deep End network was established, involving 34 general practitioner (GP) practices serving the most socioeconomically deprived populations. It aims to support the primary care workforce, explore new approaches to better support patient health and wellbeing, and advocate for changes in healthcare funding.^13^ Given the relationship between socioeconomic disadvantage and high demand for lipids modification, we explored the experiences of delivery of lipids management in Deep End practices and identified barriers and enablers to optimizing lipids management for socioeconomically disadvantaged populations.

## Method

A qualitative study was undertaken during 2021 to 2023 across the Deep End practices serving the most socioeconomically deprived patients in North East England. This design was selected to address the aim of this study exploring the experiences and factors influencing lipids management delivery in practice. Qualitative methods were deemed suitable for exploring in-depth insights and capturing the complexity and nuances of these experiences. Understanding the socio-economic context of Deep End practices was critical to addressing lipids management challenges, qualitative methods were effective in uncovering the contextual influences that quantitative methods might overlook. This flexibility was essential for capturing the dynamic and multifaceted nature of the barriers and enablers to lipid management.

Ethical approval was granted by the University of Newcastle Faculty of Medical Sciences Research Ethics Committee UK (2209/14251/2020). All data collected remained confidential to only research team members.

### Participants

The recruitment strategy drew from previous work with Deep End practices. Primary care clinicians involved in delivering and organizing lipids management who were able to provide consent were identified as eligible and invited for interviews.

Recruitment faced interruptions due to the pandemic and vaccination rollout in primary care. Besides promotion within the Deep End Network, a study poster was circulated and messages were posted on X (formerly Twitter). Convenience sampling transitioned to purposive sampling^14^ implemented considering gender, expertise, years of experience, and practice size to ensure a representative, diverse clinician sample. Interested clinicians emailed the lead author to arrange interviews.

### Data Collection

Electronic consent preceded each remote interview via Teams or Zoom per clinician preference. The lead author, experienced in health services research and qualitative methods, conducted all interviews.

A semi-structured schedule, informed by our rapid review,^9^ guided the individual interviews. Individual interviews allowed for a deeper exploration of personal experiences, thoughts, and feelings without the influence of group dynamics. Given the potential sensitivity of discussing patients’ care and their personal experiences, individual interviews provided a confidential environment where participants could speak openly and honestly. Individual interviews also helped minimize the risk of groupthink, ensuring that each person’s unique insights and experiences were captured. In addition, organizing focus groups with primary care staff could be logistically challenging while individual interviews offered a more practical solution, enhancing participation rates.

After introducing their role and practice population overview, clinicians shared perspectives on lipid assessment, management strategies, service provision, clinical pathways, implementation of current interventions targeting at-risk patients, and resources needed. They identified challenges in care delivery and support needed to optimize lipid management. With consent, interviews were recorded, allowing participants to turn off cameras. Each respondent received a £50 voucher upon completion of the interview as an incentive. This amount was carefully considered and ethically approved to ensure it was a reasonable compensation for their time and effort, without being coercive or undermining their voluntary consent. The incentive aimed to recognize the valuable contributions of the healthcare practitioners, who often had demanding schedules in Deep End practices, thus encouraging participation without exerting undue influence.

### Data Analysis

Interviews were transcribed verbatim with identifying information removed. Transcriptions were also shared with the interviewees for validation to ensure the credibility and authenticity of the data. Semi-structured interviews were employed to mitigate potential power imbalances between researchers and participants. This interview format allowed participants to express their views and experiences in their own words, creating a more balanced, and open dialog.

A framework analysis^15^ approach with 5 iterative steps was used involving all team members (a health services researcher; a clinical professor; and a public health practitioner): (1) Familiarization: YF reviewed all transcripts and JLN independently reviewed a subset to identify emerging codes, patterns, and themes. (2) Developing a thematic framework: YF and JLN compared findings to develop a preliminary coding framework, reviewed/finalized by the team. (3) Indexing: using the preliminary framework, YF coded remaining transcripts, continuously comparing data. JLN reviewed new themes and framework revisions. (4) Charting: YF summarized data with quotations into a framework matrix. (5) Mapping and interpretation: YF explored theme relationships to develop overarching themes, subsequently shared with the research team. Nvivo 12^16^ was used to manage the data and facilitate data analysis.

The context of Deep End practices, which served socioeconomically deprived populations, was integral to understanding the findings. The thematic framework guided the identification and interpretation of themes related to lipid management barriers and enablers. This study ensured trustworthiness following Schwandt et al^17^’s criteria. The study adhered to the published protocol,^18^ involved independent transcript coding by team members, and conducted regular team meetings to review emerging themes and evolving frameworks. The dependability of the findings was further enhanced through member checking, where transcriptions were validated by interviewees. Credibility was enhanced by employing semi-structured interviews that facilitated open and detailed exploration of participants’ perspectives, and the accuracy and richness of the data were achieved by member checking and independent coding by multiple researchers. Thematic framework with iterative coding and a detailed audit trail documenting all decisions and procedures ensured transparency and reliability of the findings. Reflexivity was maintained by the researchers continuously reflecting on their potential biases related to their backgrounds and roles and exchanging views on data interpretation. Verbatim transcription and direct quotations maintained the objectivity and transparency of the findings, reflecting participants’ voices authentically. Additionally, the study provided detailed contextual descriptions of Deep End practices and participant demographics, enhancing the applicability of findings to similar settings facing lipid management challenges.

## Results

Of 34 GP practices, 15 clinicians from 6 practices expressed interest and were recruited including 10 GPs, 1 GP trainee, 2 practice nurses, 1 pharmacist, and 1 physiotherapist. All were White British except 1 GP with an ethnic minority background. Experience in Deep End practices ranged from 2 to 33 years (Table 1).

**Table 1.** Characteristics of Interviewees (n = 15).

Characteristics	N (%)
Gender	
Male	9 (60)
Female	6 (40)
Job title	
GP	10 (67)
GP trainee	1 (7)
Nurse	2 (13)
Pharmacist	1 (7)
Physiotherapist	1 (7)
Ethnicity	
White	14 (93)
Non-White	1 (7)
Year of working	
1-3	2 (13)
3-6	4 (27)
6-9	5 (33)
≥10	4 (27)
Practice size (n = 6)	
<5000 patients	1 (17)
5000-10 000 patients	—
10 000-15 000 patients	3 (50)
15 000-20 000 patients	2 (33)

Themes that emerged include complex multimorbid patients with competing priorities, limited access and follow-up to supporting services, being flexible and working beyond guidelines, high workload with inadequate support, and the need for care integrity and sustainable support.

### Complex Multimorbid Patients With Competing Priorities

Interviewees described practice patients as complex with multiple CVD risk factors related to smoking, substance use, low income/education level, and poor socioeconomic status. Compared to the general population, patients often experienced early onset of multiple long-term conditions, for example, obesity, diabetes, and cancer, as well as high stress, anxiety, and depression levels.

*Comorbidities, they’ve nearly always got hypertension and diabetes or COPD or asthma, it’s unusual to have just one condition. . .Most of our patients are on lots of tablets for lots of conditions with lots of problems. People have heart attacks at 20-odd in my practice, strokes at 20 and 30, it’s not uncommon. (HP1, male GP)*

All clinicians reported that socioeconomically disadvantaged patients faced challenges prioritizing their health due to severe competing priorities including food insecurity, vulnerable living conditions, and job uncertainty. Low health literacy made it difficult to discuss CVD preventive strategies aimed at reducing risks in the next 10 years. Clinicians understood patients’ limited resources and involvement in lifestyles that hampered health prioritization.

*They[patients]’re not very bothered about their health. They don’t want to be proactive in changing their health although they’re aware of their conditions and their blood results and things not going well. (HP7, female nurse)*

*They might be worrying about whether they’re safe from people that they’re living with or they might worry that their son is about to go to prison because of something or other. (HP2, male GP)*

Consequently, patients neglected their health and sought urgent care when symptoms worsened, resulting in a cycle of complex health needs.

*If you don’t find them early on, you will find them when they’ve had their heart attacks or strokes. (HP11, female GP)*

### Limited Access and Follow-Up to Supporting Services

Clinicians reported internal and external barriers making lipids management opportunistic in deprived populations. Internal barriers included patients’ competing priorities, low health literacy, limited medical knowledge and poor cholesterol risk awareness.

*There’s still always going to be certain people that can’t access services. They’re keen to be involved but their landline is defunct, their mobile number is constantly changing because they’ve run out of money and then they get discharged from the service and then six weeks later they come back and then we have to start up again. So certainly for people who are struggling financially, getting in to see them is a little bit of a challenge. (HP10, female GP)*

Many clinicians reported misconceptions or myths perceived by patients about statins causing memory loss, dependency, and muscle pain, which reduced motivation for preventive care.

Externally, geographical, financial, and cultural factors impeded access. Patients faced difficulties travelling for services located outside the GP practice or affording healthy diets. Limited funding restricted in-house lifestyle intervention facilities, such as smoking cessation clinics.

*People in this area don’t travel out of the area as much as people elsewhere, with access to their own cars and things like that. Bringing some of that nearer would be helpful. (HP6, male GP)*

Poor patient compliance with medication, lifestyle changes, and follow-ups were also commonly reported, largely due to life uncertainties, financial constraints, and logistical challenges hindering full engagement with health services for follow-up.

### Being Flexible and Working Beyond Guidelines

All clinicians felt adhering strictly to the National Institute for Health and Care Excellence (NICE) guidelines was impossible for their socioeconomically disadvantaged patients, as the guidelines did not account for their circumstances. Consistently implementing care management plans proved challenging given patients’ routine life realities. Instead, clinicians worked flexibly, providing proactive care beyond guidelines, such as engaging younger patients for blood tests, building trust over years, extending working hours, and developing patient-driven strategies.

*You can’t follow it [guideline], I mean patients have MI [myocardial infarction] in their 20s and 30s, It’s a bit like the reason we do lipids and HbA1cs, you’d say, that person is far too young and I’m like, no, they’re exactly where they might be in my demographic. (HP1, male GP)*

*It’s [NHS Health Checks] zero use, that age [of 40] is far too high for my population. (HP9, male GP)*

### High Workload With Inadequate Support

Managing patients in deprived communities required extra resources for care management and coordination due to comorbidities and interactions between medical and social factors—a time-consuming, complex process. Clinicians needed to address social determinants through targeted interventions, education, clear communication to support condition understanding, prevention, treatment options, and self-management strategies. However high patient volumes and complex cases strained clinicians’ time, preventing comprehensive care, and potentially leading to burnout.

*My colleagues have 15-minute appointments. We would love to do that here, but then we would have to reduce the number of appointments by 50% or 33%, how would we do that, who are we not going to see. (HP4, male GP)*

Staff shortages compounded the burden of managing complex cases, making the job feel unsustainable long-term for some. Funding cuts for in-house services also made it difficult to track patients’ progress toward lifestyle modification.

*It’s a tough job which means that people don’t stay because it’s hard, people don’t follow advice, it’s a hard job. We are seriously under-resourced, the support you need is more people but that’s always a tricky thing. (HP2, male GP)*

*She [the nurse] was with us for nearly a year. We got her trained up, but then she couldn’t cope with the pressure of the job because it was too much. (HP12, female nurse)*

### The Need for Recognition, Integrated Care, and Sustainable Support

The most frequently identified need was recognition of patients’ situations and clinicians’ efforts in deprived communities. To address medical and social needs, clinicians suggested a systematic integrated approach with accessible services provided by a multidisciplinary team.

*We’ve got a mental health nurse just starting the practice and that’s a good thing because there’s an infinite amount of mental health problems with our patients, well all patients I suppose. He’s there on site so they can see him face to face so that’s a new initiative in the last four months or three months. (HP15, female GP)*

Not only more staff, but staff with adequate skills to manage the complexity of Deep End practices were needed, for example, “more practice nurse time, more healthcare assistant time.” (HP14, female GP). Accessible, sustainable training focused on different health conditions for clinicians in deprived communities was recommended, with peer learning seen as useful for sharing lipid management knowledge across roles. Clinicians acknowledged that addressing all identified needs was associated with funding issues to ensure sustainability.

*I think it’s all to do with funding really. I don’t think we can afford that [in-house smoking cessation service)]but perhaps over a PCN [primary care network], if funding was made available. (HP14, female GP)*

## Discussion

This study focused on interviewing healthcare staff in the Deep End practices to explore the delivery of lipid management, identifying barriers and enablers, and highlighting clinician needs for optimizing services for socioeconomically disadvantaged populations. Patients in these settings often present with multimorbidity, complex needs, and limited access to behavior and lifestyle modification services. Clinicians reported employing tailored approaches to lipid management but faced persistent challenges, including funding constraints, high workloads, and shortages in staffing and training, particularly for community service delivery in deprived areas. These findings underscore the urgent need for a systematic and integrated care approach that offers sustainable support to primary care in socioeconomically disadvantaged communities.

The findings of this study contribute to the existing literature in the context of NHS Health Checks^19,20^ for deprived patients. Previous studies have predominantly focused on older populations,^20^ limiting their applicability to Deep End practice where patients are typically younger and face distinct socioeconomic challenges. Despite our regional focus in North East England, the national relevance of our findings is evident. Barriers identified in this study align with those reported in studies from London,^21^ such as the lack of evidence-based interventions and limited access to essential primary care resources. Addressing these barriers holistically is crucial for improving patient outcomes across the UK.

Research consistently shows that individuals in the most deprived areas experience disparities in healthcare access, quality, and outcomes.^22^ The barriers identified in this study, ranging from competing priorities and low health literacy to poor compliance/awareness of consequences, and geographical, financial, and cultural barriers, shed light on why these disparities persist in lipids management. These factors make lipid management opportunistic rather than targeted, exacerbating inequality outside the healthcare system’s control. This adds evidence to the recent report suggesting that inequalities are most pronounced for metrics heavily influenced by factors including inadequate housing and social care shortcomings outside the NHS’s scope.^22^ Addressing these barriers requires a comprehensive approach spanning across society to tackle deprivation through education, employment, housing, and living environment.

Patients in socioeconomically deprived communities are disproportionately affected by multimorbidity, reflecting higher prevalence rates in these groups.^23^ Coupled with limited access to healthcare services, deprived populations face inadequate lipid management, thereby increasing their CVD risk and complicating multimorbidity management. Addressing lipid management is integral to achieving the NHS Long-Term Plan’s clinical priority of improving prevention, detection, and care.^8^ However clinical guidelines often lack clarity on recommended approaches for deprived populations. This study highlights a specific direction on targeted lipid management to reduce multimorbidity in primary care, aligning with calls from previous studies to address multimorbidity in diverse healthcare settings.^24^

Moreover, this study reported patients’ misconceptions and myths about statin side effects, influencing adherence rates in deprived areas. While statin prescribing rates are comparable between affluent and deprived areas,^25^ higher use is detected in deprived areas contributing to total cholesterol reduction. Efforts to increase statin use should focus on tailored interventions^9^ that include effective communication, patient education, and information provision dispelling myths about medication side effects. It is also worth noting that 1 study found financial incentives alone did not motivate statin adherence in patients.^26^ More research is needed on effective, acceptable multifaceted interventions to engage extremely socioeconomically disadvantaged communities and optimize their lipid management in primary care.

Complex health needs and multimorbidity prevalent in deprived areas place significant strain on GP services. Flexibility in care delivery and deviation from guidelines are necessary to meet these diverse needs, yet inadequate funding and staffing shortages persistently limit access and support for primary care providers. Addressing underfunding and understaffing in primary care is crucial, particularly as the recent report shows that clinical teams in deprived areas are responsible for 10% more patients with fewer fully qualified GPs since 2015.^10^ Despite promises of fairer funding, disparities persist, risking wider care inequalities. An independent review of GP funding allocations based on healthcare needs is recommended.^10^

### Strengths and Limitations

This study represents the first exploration in the UK of lipid management delivery within GP practices serving socioeconomically disadvantaged populations. The findings of this study have been fed back to the Academic Health Science Network (AHSN) Lipids & Familial Hypercholesterolemia National Program,^27^ implemented to identify patients at high risk and improve cardiovascular health. This study has guided the delivery of this program in the Deep End practices with emerging outcomes to follow.

However, the pandemic significantly affected Deep End practices,^13^ impacting recruitment and resulting in participants from only 6 out of 34 practices despite a diverse sample interviewed. Consequently, some views may be underrepresented. Nevertheless, data collected from participants converged around key emergent themes, suggesting data saturation, and achieving a thorough exploration of views/experiences.

### Implications for Research and Practice

Primary care clinicians face multiple challenges in delivering lipids management to deprived populations, including higher risks of multimorbidity and difficulties engaging due to poor health literacy, competing interests, and financial constraints. Clinicians should be willing to adapt their services, target other dimensions of social exclusion and marginalization that often accompany socioeconomic disadvantage, and develop accessible care tailored to low health literacy, competing interests, and financial constraints working with multidisciplinary teams. Continuous research is necessary to update guidance, assess the effectiveness, and cost-effectiveness of tailored interventions for lipid management, and enhance CVD outcomes among those with low socioeconomic status.
