# Corrigendum to “Lipid Management in Primary Care for Socioeconomically Disadvantaged Populations in Northern England: A Qualitative Study”

**DOI:** 10.1177/21501319241306567

**Published:** 2024-12-02

Fu Y, Sowden S, Newton JL. Lipid Management in Primary Care for Socioeconomically Disadvantaged Populations in Northern England: A Qualitative Study. *Journal of Primary Care & Community Health*. 2024;15. doi:10.1177/21501319241272026

In the above referenced article, the second author’s surname has been corrected from “Sewden” to “Sowden”. Additionally, the second affiliation has been updated and now reads as NIHR Applied Research Collaboration North East and North Cumbria, Cumbria, Northumberland, Tyne & Wear NHS Foundation Trust, UK

The referenced article has been updated online.

